# Preparation of Graphene-Perfluoroalkoxy Composite and Thermal and Mechanical Properties

**DOI:** 10.3390/polym10070700

**Published:** 2018-06-25

**Authors:** Wanlong Zhang, Haibin Zuo, Xinru Zhang, Jingsong Wang, Longfei Guo, Xing Peng

**Affiliations:** 1 State Key Laboratory of Advanced Metallurgy, University of Science and Technology Beijing, Beijing 100083, China; 18811343560@163.com (W.Z.); wangjingsong@ustb.edu.cn (J.W.); guolongfei614@163.com (L.G.); s20161311@xs.ustb.edu.cn (X.P.); 2 School of Energy and Environmental Engineering, University of Science and Technology Beijing, Beijing 100083, China; xinruzhang@ustb.edu.cn

**Keywords:** graphene, dispersion, composites, wear-resistant, thermal conductivity

## Abstract

Perfluoroalkoxy (PFA) material exhibits perfect corrosion resistance under both acid or alkaline circumstances; thus, steel heat exchangers are being substituted by those made of PFA in high corrosion atmospheres. However, the low thermal conductivity of PFA degrades its heat transfer efficiency. Based on the extremely high heat conductivity of graphene, a novel grapheme-PFA composite was proposed to simultaneously meet the demands of heat transfer and corrosion resistance. Ultrasonic dispersion technology was used to disperse the aggregated graphene in the composite. Graphene–PFA composites with different graphene contents and using different dispersing solvents were prepared with a hot pressing method, and thermal conductivity, abrasion resistance, crystallization and pyrolysis properties were investigated. The thermal conductivity of PFA composites with graphene content of 20 wt % reached 5.017 W (m·k)^−1^, which is 21.88 times that of pure PFA. The relationship between the abrasion loss and the friction coefficient of the composites with different graphene contents was obtained. A thermogravimetric analyzer was used to investigate the crystallization and pyrolysis behavior of the composites; correspondingly, the temperature range that composites work in was determined. The heat conduction mechanism was analyzed through the thermal conductivity model of composite materials. The composite material is expected to play an important role in the development of high-performance thermal equipment.

## 1. Introduction

Heat transfer materials can transfer the heat efficiently from hot fluid to cold fluid, improving the energy conversion efficiency and the cascade of energy utilization in the fields of electric heating, aerospace, chemical engineering, material science, and energy power. Metallic heat exchangers have been widely utilized in many industrial processes; however, when used in corrosive situations, their short campaign life has been a serious defect. Moreover, a heat dissipation component, characterized by high thermal conductivity, resistance to acid and alkali corrosion and anti-aging properties with a lightweight and flexible structure is widely required. Fluoropolymer is characterized by a high degree of crystallinity and high molecular weight and possesses excellent physical properties, especially for its corrosive resistance, whereas its low heat diffusion capacity hampers its application as a heat exchanging material [1,2,3,4]. Graphene, since its discovery in 2004, has drawn much attention due to its good strength, conductivity and thermal conductivity properties [5]. Therefore, in past decades, many researchers have strived to develop nanocarbon-fluoropolymer composite materials with excellent thermal conductivity, corrosion resistance, abrasion resistance and other excellent characteristics to replace some metallic and inorganic non-metallic components [6,7].

Meng et al. [8] prepared carbon nanotube (CNT)-filled perfluoroalkoxy (PFA) films using a blade-coating method to improve the electro-thermal properties; Eslami et al. [9] analyzed anisotropic heat transport in nanoconfined polyamides and proved that the interlayer spacing and porosity had very important influences on the heat conductivity; Cai et al. [10] synthesized a PTFE (polytetrafluoroethylene)–graphene composite through compression molding and free sintering and investigated the range of thermal conductivity with different graphene contents. Rooyen [11] clarified the effects of the addition of graphene and the preparation methods on the physical properties of the grapheme-PTFE composites; Tanaka et al. [12] investigated the friction and wear properties of two kinds of PTFE composites filled with CNTs and graphene, respectively, and they thought the lamellar structure of the graphene enhanced the self-lubricity of the composite due to the sliding between the graphene sheets; Takeichi [13] and Sidebottom [14] also executed relative research on the friction properties of carbon-PTFE and PFA-alumina composites. Additionally, PFA-CNTs and PFA-silver composites were synthesized as super-hydrophobic coating materials by Wang [15] and Zhai [16] et al. Previous research has proved that the nanocarbon-fluoropolymer composite possesses outstanding properties, and the melt processability gives the PFA advantages in industrial production and recycling utilization. Graphene also has been a major selection to improve the properties of fluoropolymers. However, the investigation of grapheme-PFA composites has been less reported, and the preparation method of the composites has not yet been perfected, especially in the aspects of nanomaterial dispersion and the molding process. The uniform dispersion of graphene in the thermal conductive substrate is a determining factor in the development of high-performance thermal conductive materials.

In this study, grapheme-PFA composite was prepared, and the properties of thermal conductivity and abrasion resistance were investigated emphatically. Considering the strong aggregation tendency of graphene caused by strong van der Waals forces and π–π stacking between graphene layers [17], the ultrasonic method [18,19,20,21,22,23] was adopted to disperse the graphene in a water-alcohol solution with different ratios of water to alcohol. The effects of ultrasonic treatment and surface tension of solution on dispersion were discussed deeply. Different from compression molding and free sintering, the hot pressing method was performed in our experiments, which made it easier to achieve continuous production. 

## 2. Experiment

### 2.1. Materials

**Figure 1:**
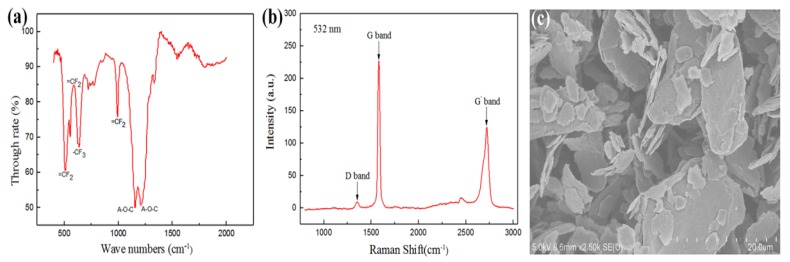
Characterization of materials: (**a**) infrared spectrum of PFA; (**b**) Raman spectrum of graphene; (**c**) SEM image of graphene.

The PFA powder, supplied by Tenghai Engineering Plastics (Guangdong, China), was modified PFA, with a density was 2.16 and an average particle size of 21.816 μm. The infrared spectrum of PFA is shown in Figure 1a.

The graphene nanosheet (GT-G03) was supplied by Kaina Graphene (Xiamen, China). The graphene layers were only 1 to 3 (manufacturer’s test report), and the water content was 1.08%. The average diameter (average equivalent diameter of graphene measured by laser particle size method), and bulk density of the nanosheet were 8.96 μm and 0.04 g/cm^3^, respectively. The Raman spectrum is shown in Figure 1b. The 2D/G ratio and half width of the 2D peak indicated few layers of graphene [24,25,26]. Figure 1c presents the scale-like morphology of the graphene nanoclusters which were observed by field emission scanning electron microscopy. 

The alcohol with 99.97% purity was supplied by the Sinopharm Group (Shanghai, China). The density was 0.79 g/mL.

### 2.2. Dispersion Method

Graphene sheets are easily aggregated due to the extremely strong van der Waals forces and π–π stacking and formed multi-layered graphene platelets (Figure 1c). It has been reported that the properties of graphene material resemble those of graphite. However, when the number of graphene layers is over 10, it cannot exhibit the excellent properties of graphene sheets [27]. 

Ultrasonic treatment technology is often utilized to improve the dispersion effect of micro-particles in dispersions. In this study, the optical density of the dispersion liquid was used to evaluate the dispersion effect. The optical density of the dispersions was detected by spectrophotometer (TAS-990) immediately after the ultrasonic treatment was accomplished. Firstly, the dispersion effects of graphene in alcohol after ultrasonic treatment with different amounts of power and treating times were compared to obtain the optimal operation parameters. After that, the effects of the surface tension of dispersions were investigated. Dispersions with specific surface tensions were prepared through mixing alcohol and water at certain ratios. The surface tension of the dispersions was measured by the pendant drop method; the measurement results are listed in Table 1. The graphene concentration of all dispersions measured above was 2 mg/mL.

### 2.3. Preparation of Composite Materials

**Figure 2:**
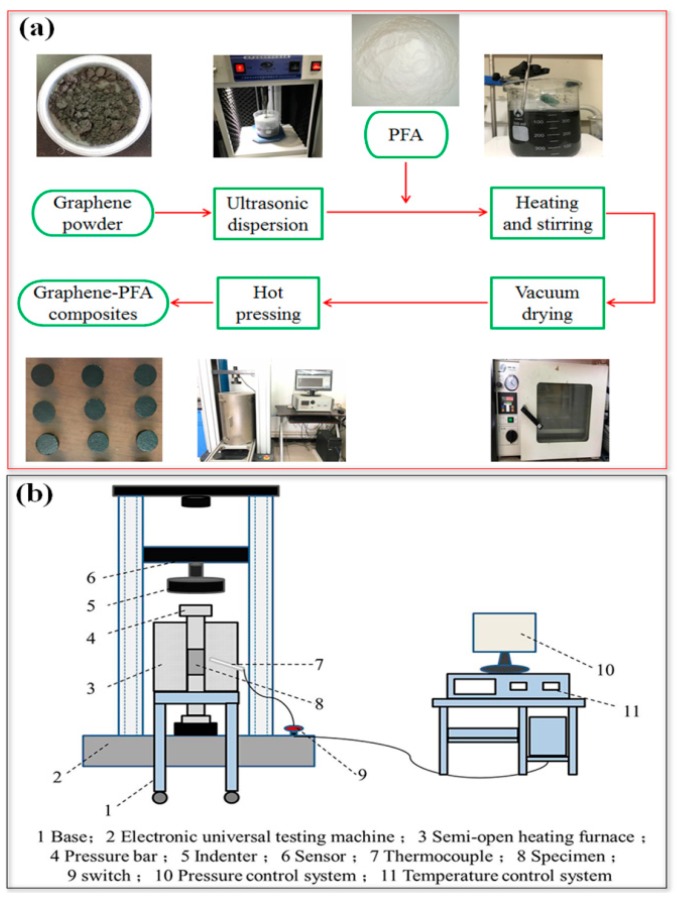
Preparation of graphene-PFA (perfluoroalkoxy) composites: (**a**) schematic flowchart of preparation; (**b**) hot pressing device.

In order to minimize the effect of water on the experimental results, graphene and PFA were dried to remove moisture before the experiments. The preparation of graphene-PFA composites is shown in Figure 2a. In Step 1, the graphene was added to the dispersion and sonicated for 2 h using a sonicator at a power of 300 W. Then, a certain amount of PFA was added to the graphene dispersion, stirring thoroughly to achieve uniformity. The amounts of graphene and PFA used in the experiment are shown in Table 2. In Step 2, the dispersion was placed on an electromagnetic heating stirrer with a speed of 800 r/min at 60 °C. When only a small amount of dispersion remained in the beaker, the sample was transferred to the vacuum drying oven and dried for 24 h at a temperature of 60 °C. The dried graphene-PFA powder was subsequently removed from the beaker for grinding. In Step 3, 3 g of the powder was loaded into a mold with an inner diameter of 30 mm. Different from the cold-pressed sintering, in the experiment, the powder was hot-pressed at a temperature of 380 °C with a pressure of 12.7 MPa for 2 min, as shown in Figure 2b. Then, the mold was taken out from the oven and cooled down in air. Five graphene-PFA composites with different graphene contents (1, 5, 10, 15, and 20 wt %) were prepared, and the properties of the composite were investigated. The weight of each sample was 5 g.

### 2.4. Composite Characterization

The fracture surface morphology of the graphene-PFA composite material was observed with a scanning electron microscope (SEM) (Hitachi SU8010, Tokyo, Japan) to determine the distribution of the graphene in the PFA. The morphology and layers of graphene were observed by transmission electron microscopy (TEM) (Hitachi HT7700, Tokyo, Japan).

**Figure 3:**
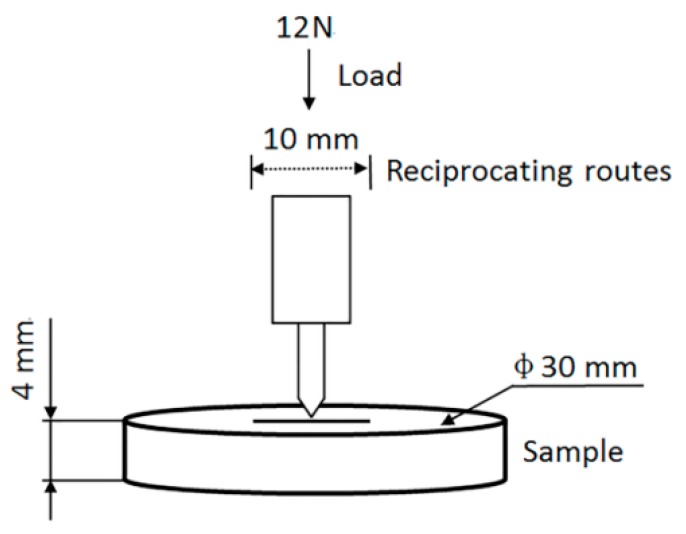
Schematic diagram of friction and wear testing.

The friction and wear properties of the composites were tested through a reciprocating friction and wear testing machine; the schematic diagram is shown in Figure 3. The composite material was fixed on a test bench, 30 mm in diameter and 4 mm in thickness. Then, a constant load of 12 N was exerted on the sample. The friction probe was made of chrome steel with a reciprocating frequency of 2 Hz and a reciprocating stroke of 10 mm; the test time was 35 min. 

The thermal stability of the graphene–PFA composites was investigated by thermogravimetry and differential scanning calorimetry (DSC) (DTA, DSC, X70, Bochum, Germany). A sample of approximately 15 mg was loaded into a crucible and then heated from 50 to 800 °C at a rate of 10 °C/min in an argon atmosphere and then immediately cooled from 800 to 50 °C at the same rate.

The thermal conductivity of the composites was detected by the Hot Disk method (Hot Disk, TPS 2500S, Gothenburg, Sweden) to ascertain the effects of the different dispersions and different graphene dosages on the thermal conductivity of the composites.

## 3. Results and Discussion

### 3.1. Dispersion of Graphene

**Figure 4:**
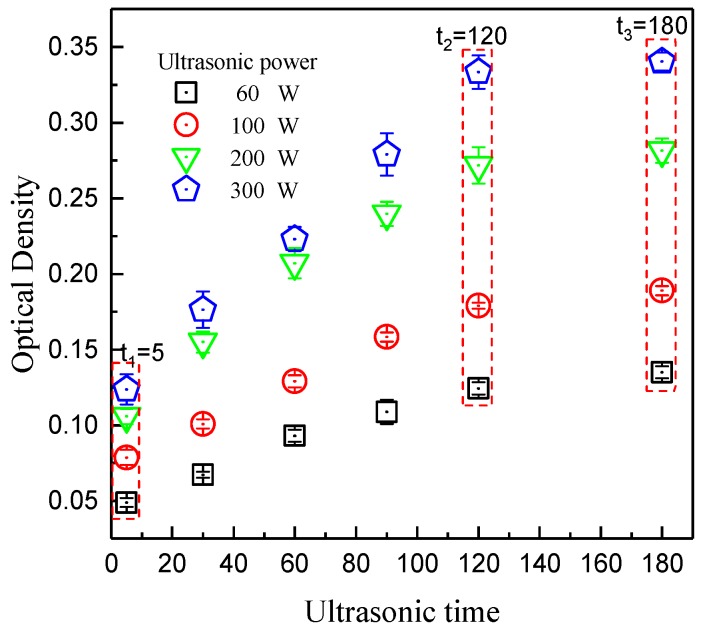
Range of optical densities of dispersions with ultrasonic power and treating time.

**Figure 5:**
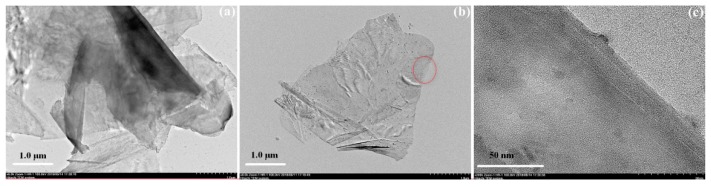
TEM images of graphene after different ultrasonic treatment times: (**a**) 5 min; (**b**) 60 min; (**c**) magnification of red circle marked area of (**b**).

Avoiding the agglomeration of graphene in PFA is a critical step for obtaining a good performance composite. The optical density of dispersions was detected to indicate the dispersion effect of graphene—the higher the optical density was, the better the graphene dispersed. Figure 4 shows the varieties of optical density with ultrasonic time and power. The optical density was improved as the ultrasonic treatment time and power increased. When the treating time was greater than 120 min, the optical density did not grow obviously, meaning the graphene had been dispersed adequately. Therefore, the treatment time was set at 120 min. Under this condition, the promotion of ultrasonic power always had an obviously positive effect, so we set the power to 300 W. Although improving the power further might have been more effective, it would also cause more energy consumption. The TEM pictures of graphene after dispersion by the sonication method are listed in Figure 5. When the ultrasonic treatment time was only 5 min, graphene presented a multilayered structure, as seen in Figure 5a. When the time of ultrasonic treatment was prolonged to 60 min, the amount of graphene with structures with few layers, as seen in Figure 5b, increased. The edge of the graphene sheet, which is marked by red circle in Figure 5b, is enlarged and shown in Figure 5c. In the picture, graphene with few layers is clearly presented. Thereby, the sonication method could improve the dispersion effect of graphene, resulting in good connectivity between graphene compounds.

**Figure 6:**
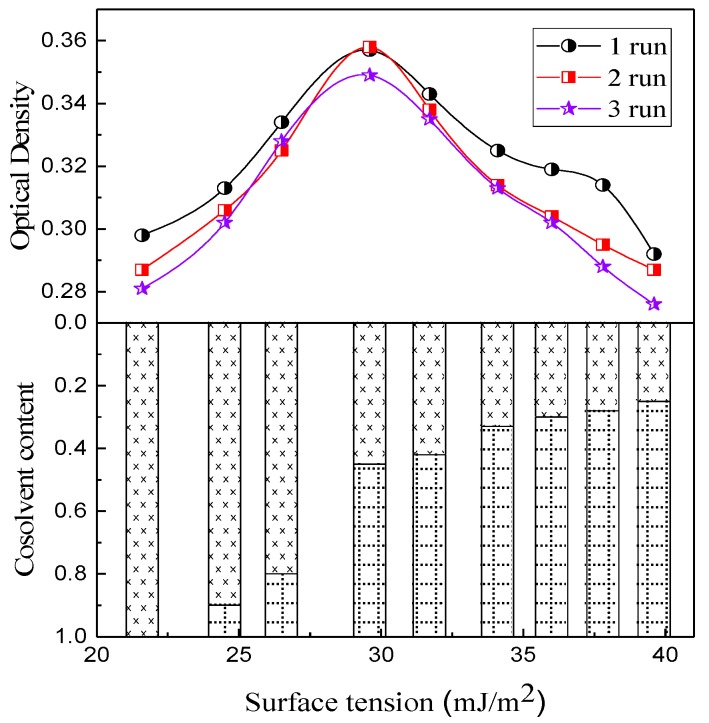
Range of optical densities of dispersions with surface tensions of dispersions.

The properties of dispersions, especially the surface tension and wettability to graphene, are important factors for dispersion and for avoiding the re-agglomeration of graphene after complete dispersion. The surface tension of pure alcohol was only 21.6 mJ/m^2^, far smaller than that of the pure water, which was 73.1 mJ/m^2^. Therefore, alcohol exhibited a better wettability than water. Figure 6 shows the evolution of the optical density of dispersions with surface tension under the conditions of 300 W power and 120 min treating time. The highest optical density was obtained when the surface tension of dispersions was 29.6 mJ/m^2^ and the average value of three runs was 0.35. A higher or lower surface tension broke the equilibrium of forces between the micro-particles and liquid molecules, causing the agglomeration of graphene; thus, the optical density decreased.

Consequently, the dispersions with a surface tension of 29.6 mJ/m^2^ weres used in the subsequent experiments (the volume ratio of alcohol to water was 45:55), and the graphene dispersion was sonicated for 120 min using a power of 300 W.

### 3.2. Thermal Decomposition Characteristics of Composite

**Figure 7:**
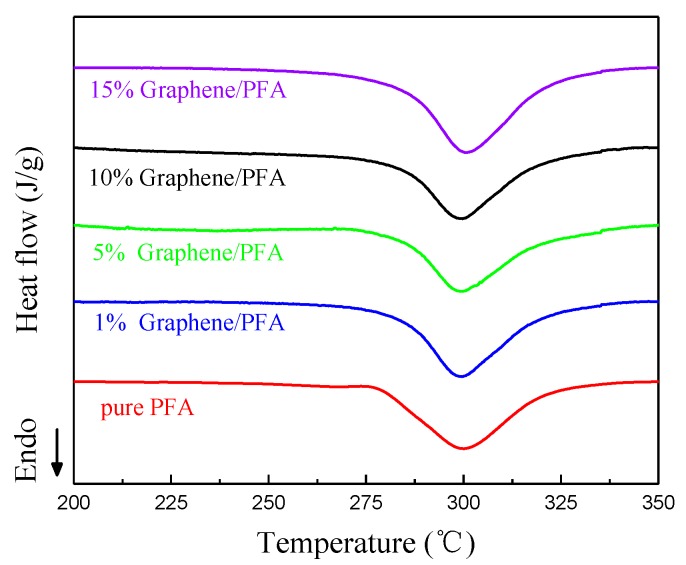
DSC (Differential scanning calorimetry) curves of the graphene-PFA composites.

In order to study the influence of the graphene content on the crystallinity of the PFA matrix, DSC was used to analyze the melting behaviors of the composites. Figure 7 shows the enthalpy changes of the graphene-PFA composites with different graphene contents with increasing temperatures. It is evident that the melting peak area of pure PFA material was the largest and the melting peak area enlarged with a rise in the amount of graphene, indicating that the melting enthalpy (Δ*H*_m_) of the composites tended to increase. Table 2 lists the melting temperature, melting enthalpy and crystallinity degree of PFA with different amounts of added graphene.

The melting temperatures (*T*_m_) of the five samples were similar and ranged from 299 to 301 °C. Clearly, compared to pure PFA, the melting enthalpy decreased slightly when 1% graphene was added, while improving the graphene contents further from 1% to 15% increased the melting enthalpy gradually. The crystallinity (*X*c) of the composites was calculated using the formula, *X*c = *H*m/(*W*_PFA_ × *H*_m_*), where Δ*H*_m_* is the melting enthalpy of 100% crystalline PFA and *W*_PFA_ is the mass fraction of the PFA [28]. The crystallinity degree was affected by the number of nucleation sites and migrating ability of the crystalline substance. The crystallinity degree of the PFA decreased slightly when a small amount of graphene was added to the pure PFA. The graphene had a two-dimensional planar structure, although the added graphene provided nucleation sites for the PFA, which was beneficial for improving the crystallinity degree. At the same time, the graphene restricted the migration and diffusion of the polymer segments, preventing the crystallization of the composite. When the graphene content was low, for example, 1%, the effect of improving the nucleation sites was weaker than the drawback of hindering migration, resulting in a lower crystallinity degree for the PFA composite compared to the pure PFA. When more graphene was added into PFA, more nucleation sites were provided and became a dominant factor of crystallinity. The PFA close to the graphene crystalized and grew via a heterogeneous nucleation mechanism, contributing to a slight increase in crystallinity. However, promoting the graphene content further, the PFA nucleation sites were relatively saturated and the negative role of preventing crystallinity gained the upper hand again, leading to a slight decrease in the crystallinity degree. The fluctuation in the crystallinity degree differed from results that Meng et al. [9] reported, in their research. The crystallinity degree showed a linear increasing trend with CNTs embedded in PFA. This was attributed to the difference between CNTs and graphene. The CNTs had a 1D nucleation surface with a large aspect ratio and each CNT provided multiple nucleation sites; therefore, the effect of increasing the number of nucleation sites always had an advantage over the effect of hindering migration.

**Figure 8:**
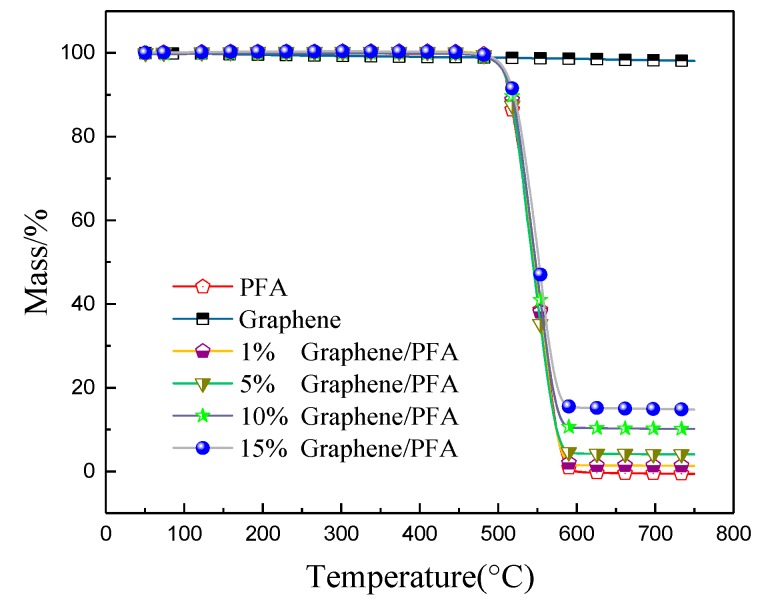
TG (Thermogravimetric) curves of the composites with different graphene contents.

The thermogravimetric (TG) curves of pure PFA, graphene and five composites are shown in Figure 8. The graphene sample had no weight change as the temperature increased. For the composites and pure PFA, there were the same weight loss features, including the starting and ending temperatures for weight loss as well as the weight loss rate, illustrating that the graphene did not affect the thermal stability of the composites. The composites had no weight loss below 480 °C. When the temperature was higher than 480 °C, the composites began to decompose and volatize. The weight of samples decreased rapidly until the temperature was about 575 °C. The residual weight exactly equaled the weight of the added graphene, which indicated that the PFA decomposed completely from 480 to 575 °C. Zhao et al. [8] also investigated the thermal decomposition characteristics of PFA–CNTs composites and proved that the additive had no effect on the pyrolysis of PFA, indicating the high thermal stability of PFA.

**Figure 9:**
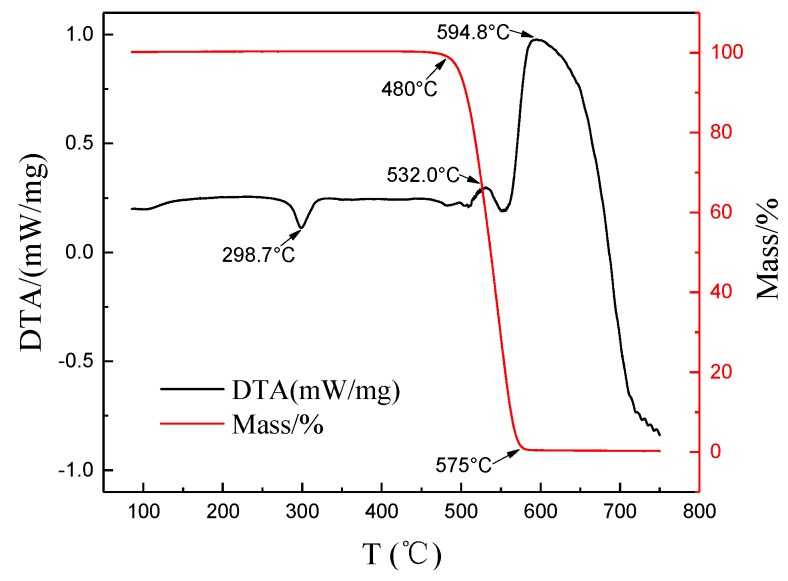
DSC curve of PFA during pyrolysis.

Figure 9 shows the DSC curves of the PFA during pyrolysis. The endothermic peak appeared at 298.7 °C, where the PFA melted. The exothermic peaks appeared at 532.0 and 594.8 °C, where the decomposition and oxidation reactions occurred simultaneously; the PFA decomposition absorbed a small amount of heat, and the oxidation released a lot of heat. No phase change occurred below 300 °C, indicating that graphene-PFA composites had a high thermal stability below 300 °C.

### 3.3. Abrasion Resistance

**Figure 10:**
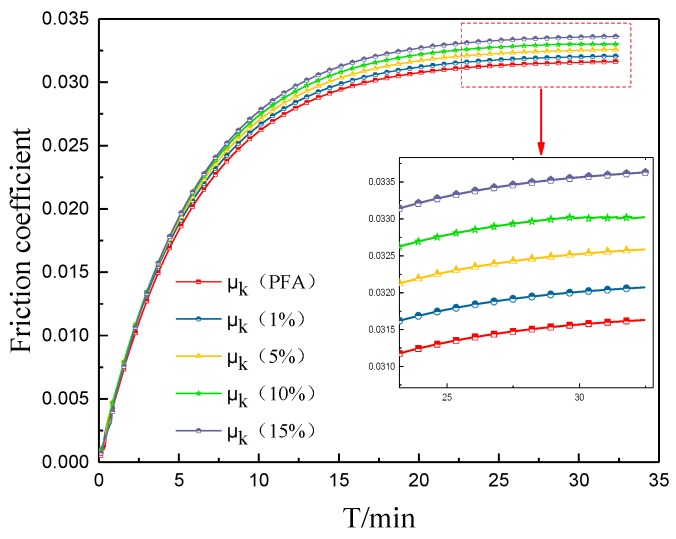
Friction coefficients of the composites with different graphene contents.

Figure 10 shows the changes in the friction coefficients of the composites with different graphene contents. The friction coefficient curves presented a parabolic shape and stabilized at the end of the test period. The friction coefficient of the composite gradually increased with a rise in the amount of graphene; the tribological properties of the composites gradually deteriorated. The friction coefficient of pure PFA was the smallest, only 0.0313; and the largest value was obtained when the graphene content was 15%, which rose up to 0.0334. The graphene that was exposed to the surface improved the roughness of the contacting area, which boosted the friction force, resulting in an increase in the friction coefficient of the composite materials [29]. 

**Figure 11:**
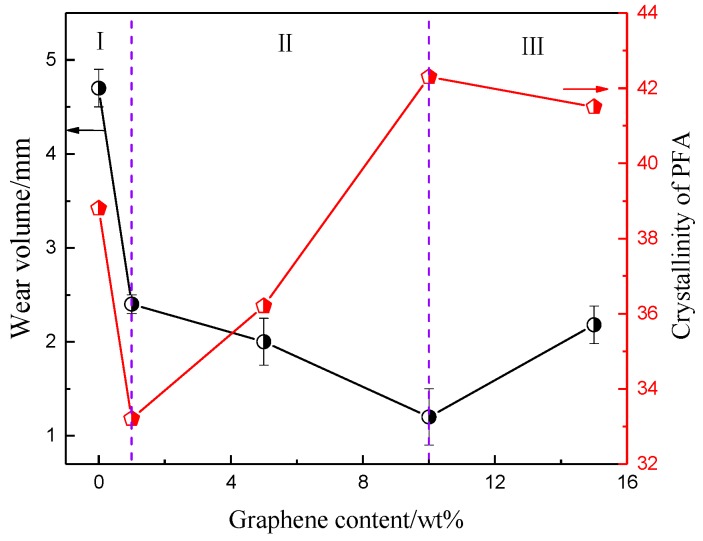
Wear resistance of composites with different crystallinities.

Figure 11 shows the wear volume and corresponding crystallinity degree of the composites with different graphene contents. The wear volume of the composites firstly decreased and then increased with the addition of the graphene but was always lower than pure PFA. In regions I and II, the graphene content was relative low, the wear volume tended to decrease and the decreased rate was more rapid in region I, indicating that the self-lubricating properties of graphene had major effects. Under the action of friction force, slipping among graphene sheets occurred and formed an effective transfer film and attachment film, which enhanced the strength and toughness of composite, ultimately resulting in the reduction of fatigue cracks and subsurface substance shedding. This has also been proved by Xia [11,30]. With an increase in the amount of graphene in region II, the rate of wear volume decreased and the crystallinity of the composite gradually increased, which resulted in greater toughness. However, the increase in added graphene caused aggregation easily in the matrix and the uniformity of the composition of the material decreased, leading to weak region appearance and a reduction of crystallinity degree. The toughening effect of the graphene on the PFA dwindled, and the corresponding wear mechanism also changed. For region III, this phenomenon was even more serious—the abrasion resistance degraded, and the wear volume increased again. Additionally, the composite material peeled itself from the matrix and formed debris during the wear; a small amount of the debris remained on the surface of the matrix, thus exhibiting an interaction between plowing and adhesive wear. Some research has proved that adhesive wear is the major type of wear for graphene-enhanced materials [12,31].

### 3.4. Thermal Conductivity

**Figure 12:**
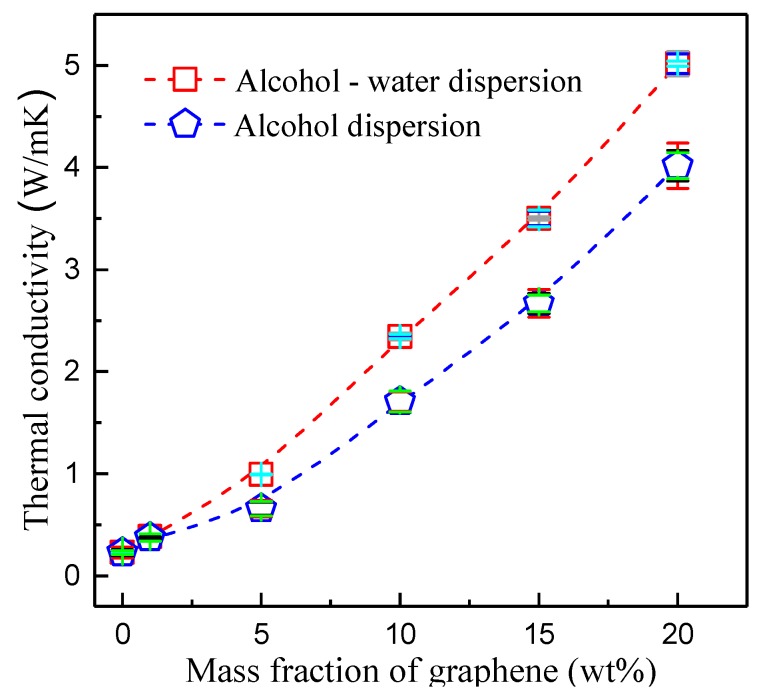
Range of thermal conductivities with graphene contents using different dispersions.

Besides improving the mechanical properties, another important role of nanocarbon material addition is to promote the electro-thermal properties. Figure 12 shows the thermal conductivities of composites with different graphene contents. In experiments, two kinds of solution, pure alcohol and an alcohol-water mixture with a ratio of 45:55, were applied to disperse the graphene. The thermal conductivity of pure PFA was relatively low—it was about 0.23 W/(m·K). With a greater graphene content, the thermal conductivity of the composites increased gradually using either pure alcohol or alcohol-water as dispersions. We also found that the thermal conductivity of the alcohol-water solution was still higher than that of alcohol, corresponding to the optical density and demonstrating the determinative role of the dispersion effect on the thermal conductivity. The maximal average thermal conductivity was obtained when the graphene content was 20 wt %. It reached 5.017 W/(m·K) and was 21.88 times that of pure PFA, which was attributed to the high thermal conductivity of graphene. According to the result, it could be deduced that the optical density of dispersions is the dominant factor in the thermal conductivity of a composite. As shown in Figure 4, there is a positive relationship between the optical density and the ultrasonic time and power, meaning that ultrasonic treatment could promote the thermal conductivity of composite through improving the dispersion effect of graphene.

**Figure 13:**
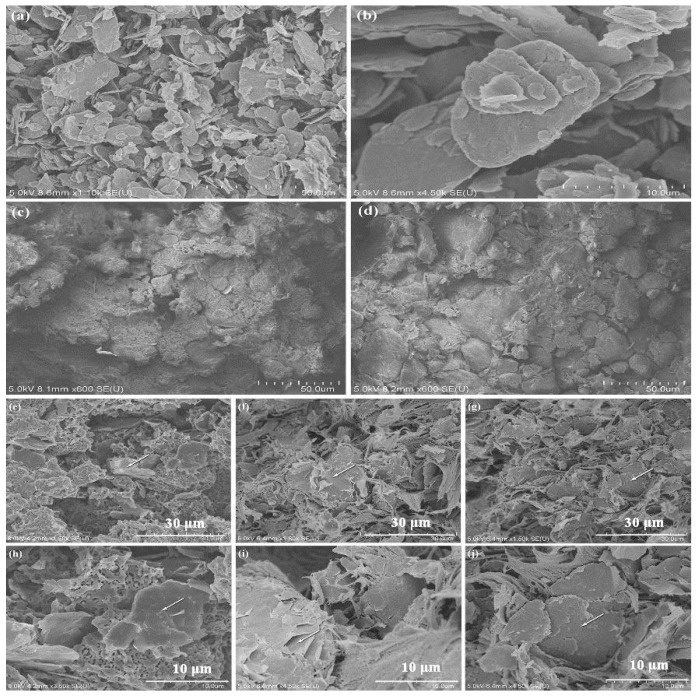
SEM images of the composites with different graphene contents: (**a**,**b**) graphene; (**c**,**d**) composites with 1 and 5 wt % graphene contents; (**e**–**g**) composites with 5, 10 and 15 wt % graphene contents; (**h**–**j**) enlarged images for (**e**–**g**).

Figure 13 shows the field emission scanning electron micrographs of the different composites. Figure 13a,b depicts the pattern of graphene that was used in the experiments. The graphene was 5–20 μm in range of particle size and presented a nanoscale lamellar structure. The unique two-dimensional planar structure contributed to excellent heat conduction. As stated above, the thermal diffusivity of pure PFA was very low, and the graphene-PFA interface also had great thermal resistance. Therefore, the filler played a dominant role in the thermal conductivity of the composite material. Compared with Figure 13c,d, the amount of visual graphene in Figure 13d is obviously higher than that in picture (c), corresponding to the original graphene content in the composites—1 and 5 wt % in (c) and (d), respectively. The particle size of PFA was larger than that of graphene. The graphene was distributed in the middle of the PFA or in the form of aggregates, forming thermal conduction paths. The white arrows in Figure 13e–j refer to graphene sheets that were agglomerated or distributed in the middle of the PFA. Consequently, the thermal conductivity of the composite material improved. Figure 13e–g show the cross-sectional images of the hot-pressed graphene-PFA composites with 5, 10, and 15 wt % graphene contents. PFA particles were in tight contact with graphene slices, and with the graphene with lamellar scale structure that established many stable high thermal conduction paths in the composite material. As the graphene content increased from 5 to 15 wt %, the thermal conduction paths in Figure 13g obviously increase relative to those shown in Figure 13e; therefore, the thermal conductivity of the composite material was greatly improved.

**Figure 14:**
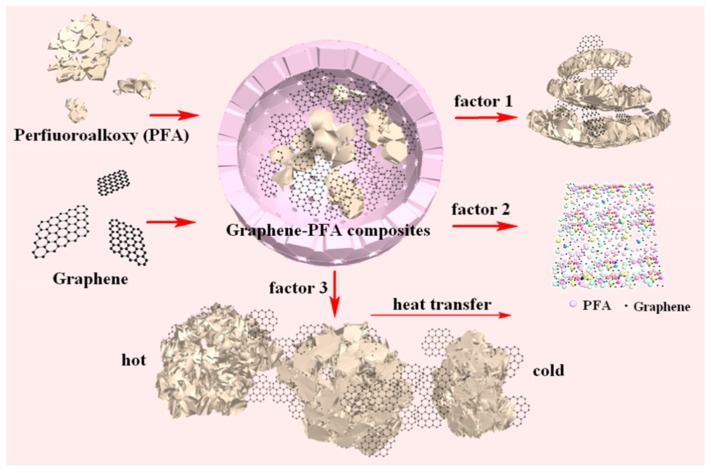
Thermal conductivity schematic of graphene-PFA composites.

According to the evolution of thermal conductivity of the composites with graphene contents shown in Figure 12 and the SEM observations shown in Figure 13, three factors were considered to affect the thermal conductivity of graphene-PFA composites—layer spacing between graphene with PFA, the porosity of graphene-PFA composites and the connectivity between graphenes. Figure 14 shows the thermal conductivity scheme of the graphene-PFA composite. Good dispersion of graphene definitely played a critical role in the thermal conductivity of the composite. Factor 1 shows the layer spacing between graphene and PFA, factor 2 presents the porosity of the composite and factor 3 indicates the connectivity of the heat conduction path formed by the graphene. Decreased interlayer spacing and porosity and the formation of good connectivity between graphene compounds could reduce the interfacial thermal resistance between graphene and PFA and phonon scattering, contributing to a rise in the thermal conductivity of the composites [14,15,16]. In the experiment, an increase in graphene content and the ultrasonic treatment were beneficial to graphene dispersion in the composite, improving the connectivity between graphene compounds. The hot pressing method favored a reduction in layer spacing of the composite. The generated bubbles during heating were easy to release under the role of high compression stress. Therefore, the thermal conductivity of the graphene-PFA composites was greatly improved. 

## 4. Conclusions

The graphene dispersion in the PFA is the most critical factor affecting the thermal conductivity of a composite. Ultrasonic treatment and proper surface tension of dispersion can promote the effective dispersion of graphene. The optimum dispersions of the graphene were composed of 45% alcohol and 55% water, with a surface tension of 29.6 mJ/m^2^. The ultrasonic power and processing time were 300 W and 120 min, respectively. 

The melting temperatures of all prepared composites stabilized in the range of 299–300 °C, and there were never decomposition or phase transition occurrences below 300 °C. Thus, a high thermal stability was exhibited. The graphene was able to provide heterogeneous nucleation and crystallization sites for the PFA, but a large amount of fine graphene limited the diffusion and migration of the PFA segments, leading to a decrease in crystallinity. 

The friction coefficient of the composites increased with the addition of graphene. The transfer films and attached films of the self-lubricating graphene reduced the wear volume of the composites, improving the abrasion resistance. When the graphene content was higher than 10 wt %, the wear volume increased slightly due to the appearance of weak regions and the reduction in the crystallinity degree caused by excessive graphene. 

The thermal conductivity of the composite improved effectively by increasing the graphene content in PFA. The maximal thermal conductivity of PFA composites reached 5.017 W/(m·K) when the graphene content was 20% by weight, meeting the demands of many industrial applications. The SEM images of the composite materials indicated that the scaly substance increased when the graphene content increased; the graphene distribution in the PFA matrix also improved. The dispersed graphenes formed a scale-like structure and provided a large number of thermal conduction paths; therefore, the thermal conductivity was enhanced. Through hot pressing, the interlayer spacing and porosity decreased, favoring the reduction of the interfacial thermal resistance between graphene and PFA. Ultrasonic treatment improved the connectivity of graphenes, reducing the phonon scattering, resulting in good thermal conductivity of the composites.

## Figures and Tables

**Table 1:** Properties of alcohol solutions with different concentrations.

Alcohol/Water (*vol*/*vol*)	Surface tension (γ) (mJ/m^2^)	Density (ρ) (kg/m^3^)
100/0	21.6	789.0
90/10	24.5	809.9
45/55	29.6	903.9
30/70	36.0	935.3
20/80	42.7	956.2
10/90	51.2	977.1
0/100	73.1	998.0

**Table 2:** Melting temperature, melting enthalpy and PFA crystallinity of composites.

Sample	Melting temperature *T*_m_ (°C)	Melting enthalpy Δ*H*_m_ (J/g)	Crystallinity degree *X*_c_ (×100%)
Pure PFA	300.7	38.8	38.8/ΔH*_m_
1 wt % Graphene	299.6	32.9	33.2/ΔH*_m_
5 wt % Graphene	299.7	34.4	36.2/ΔH*_m_
10 wt % Graphene	299.7	38.1	42.3/ΔH*_m_
15 wt % Graphene	300.6	35.3	41.5/ΔH*_m_
